# Primary Gastric Mucosal Melanoma: A Rare Etiology of Iron Deficiency Anemia

**DOI:** 10.7759/cureus.38668

**Published:** 2023-05-07

**Authors:** Usama Abu-Heija, Mohammad Darweesh, Damir Kusmic, Mark Young

**Affiliations:** 1 Internal Medicine, East Tennessee State University, Johnson City, USA; 2 Medical Education, Jordan University of Science and Technology, Ar Ramtha, JOR; 3 Gastroenterology, East Tennessee State University, Johnson City, USA

**Keywords:** primary gastric malignant melanoma, iron deficiency anemia (ida), small bowel capsule endoscopy, video capsule endoscopy (vce), mucosal malignant melanoma

## Abstract

Iron deficiency anemia is a concerning finding, particularly in males and post-menopausal females, and can have numerous underlying causes. When evaluating potential sources of gastrointestinal blood loss, bidirectional endoscopy is often necessary. We report the case of an 89-year-old female with multiple comorbidities, including atrial fibrillation treated with apixaban, who presented with symptomatic iron deficiency anemia. Extensive dermatological and radiological assessments ruled out a primary source, and subsequent endoscopy identified a rare etiology: primary gastric mucosal melanoma. This case highlights the importance of thorough evaluation in identifying uncommon causes of iron deficiency anemia such as unsuspected malignancies, hereditary conditions, and different autoimmune conditions amongst other etiologies.

## Introduction

Iron deficiency anemia (IDA) is always a worrisome finding in males and post-menopausal females, as malignancy is always a primary differential among more benign etiologies such as nutritional causes and celiac disease [1]. According to the American Gastrointestinal Association guidelines when assessing for IDA in the aforementioned population, bidirectional endoscopy should be performed and is superior to upper endoscopy alone [2].

Primary gastric malignant melanoma is an extremely rare diagnosis, especially when compared with the incidence of melanoma that is metastatic to the gastrointestinal tract (1-4%) [3]. When classifying the disease, we can use two main broad categories, cutaneous melanoma and mucosal or extracutaneous melanoma, which accounts for 1% of all diagnosed melanomas [4].

We are reporting a rare entity that presented with IDA in an 89-year-old female patient on chronic anticoagulation and was diagnosed by different endoscopic and imaging modalities.

This report was presented as an abstract at the American College of Gastroenterology (ACG) 2022 Annual Scientific Meeting, held in Charlotte, North Carolina, United States from October 21-26, 2022.

## Case presentation

**Figure 1:**
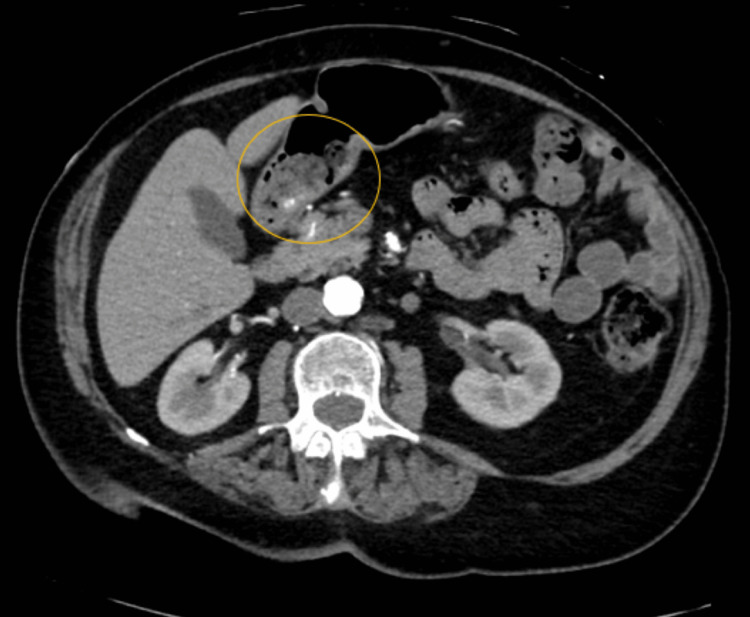
CTA of the abdomen: questionable small focus of hemorrhage in the posterior gastric antrum. CTA: computed tomography-angiography

An 89-year-old female with multiple comorbidities, including atrial fibrillation on apixaban, presented with fatigue, weakness, and exertional dyspnea. Laboratory investigations in the emergency department revealed IDA with a hemoglobin level of 8.0 g/dL. The patient denied any overt gastrointestinal bleeding at that time. Subsequently, a computed tomography-angiography (CTA) of the abdomen (Figure 1) revealed a questionable small focus of hemorrhage in the posterior gastric antrum. The patient was started on intravenous proton pump inhibitor (PPI) therapy and underwent bidirectional endoscopy after receiving adequate transfusions.

**Figure 2:**
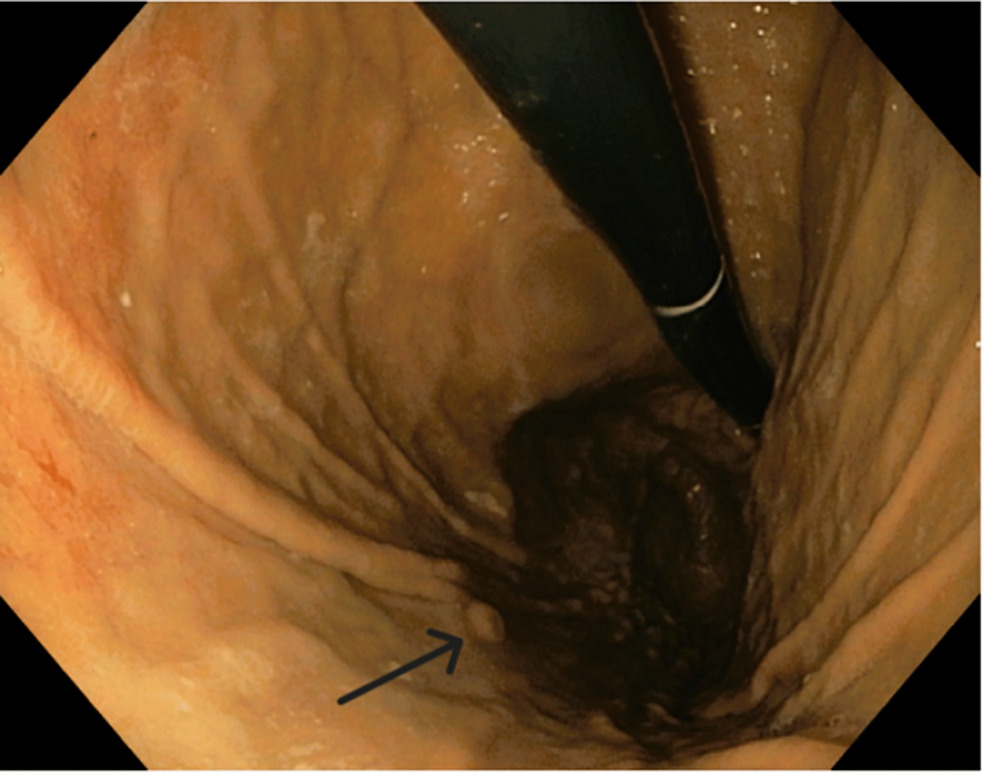
Esophagogastroduodenoscopy findings: 3-4 mm raised umbilicated gastric body lesion, removed by cold forceps.

**Figure 3:**
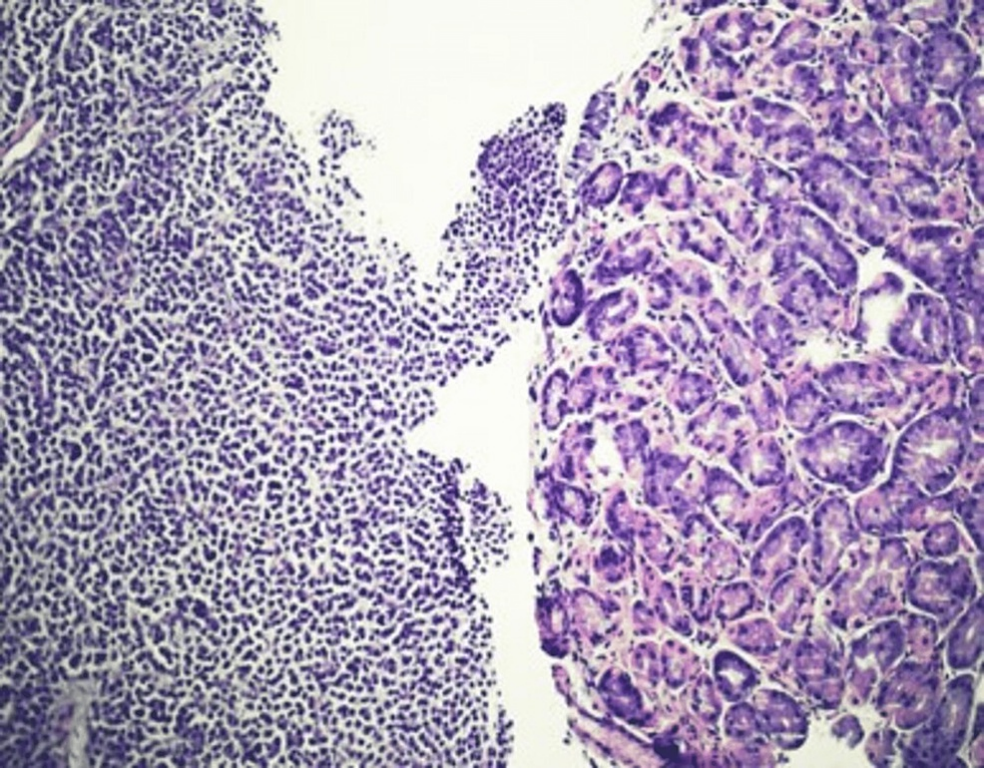
Histological slides findings: H&E of the stomach polyp shows markedly pleomorphic malignant cells that are positive for SOX-10, MART1, HMB45, and S100, consistent with the diagnosis of melanoma. H&E: hematoxylin and eosin staining

**Figure 4:**
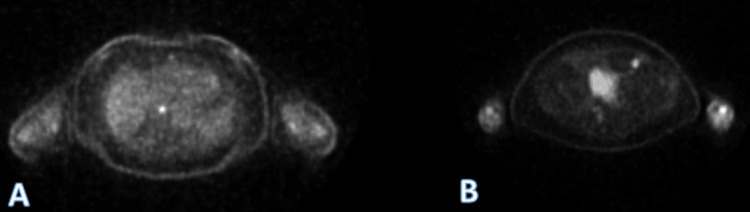
PET scan: (A) Focal uptake in a distal esophageal lymph node; (B) Focal uptake in a loop of small intestine in the left lower abdominal quadrant. PET: positron emission tomography

Endoscopy revealed a 3-4 mm raised, umbilicated gastric body lesion (Figure 2), which was removed with cold forceps. Biopsy of the gastric lesion revealed a mucosal melanoma that tested positive for SOX 10, MART 1, HMB45, and S100 (Figure 3). Further evaluation with a positron emission tomography (PET) scan for staging (Figure 4) showed focal uptake in a distal esophageal lymph node and a loop of the small intestine in the left lower abdominal quadrant.

**Figure 5:**
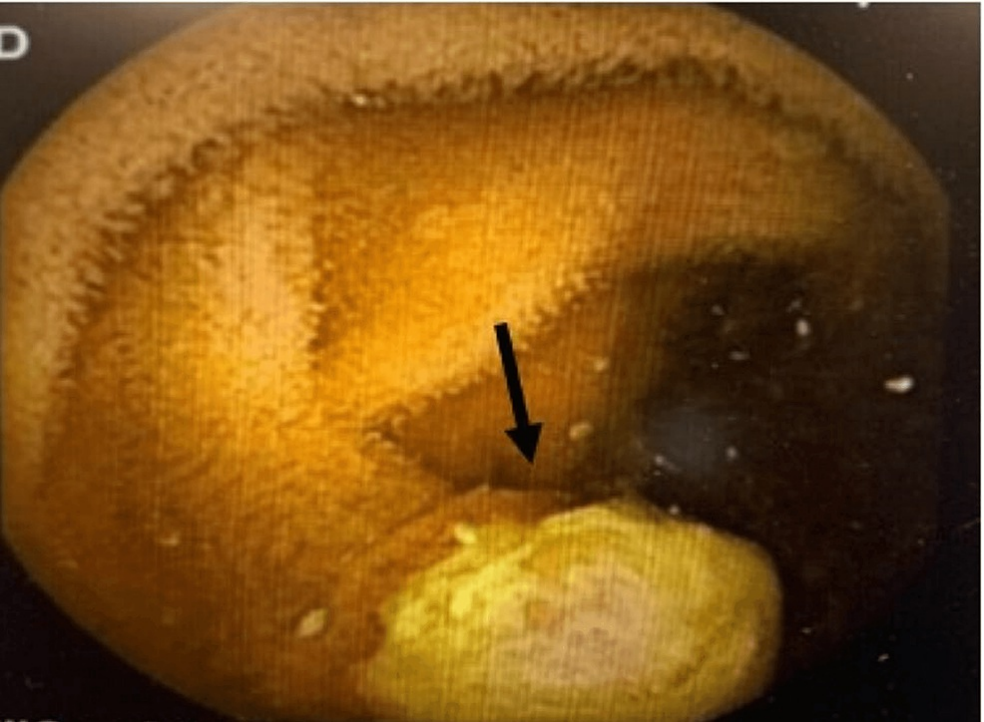
VCE findings: One of three small intestinal (likely jejunal) masses found on VCE; it had a similar mucosal pattern to the identified gastric lesion. VCE: video capsule endoscopy

To assess for a primary source, the patient underwent complete dermatological and ophthalmological evaluations, as well as a head CT scan with contrast, which did not reveal any primary lesions. In addition, video capsule endoscopy was performed to evaluate the PET uptake in the small intestine, revealing three small intestinal masses (likely jejunal) with a similar mucosal pattern to the identified gastric lesion (Figure 5). The patient was referred to oncology and eventually underwent three rounds of radiotherapy followed by systemic chemotherapy.

## Discussion

When assessing for IDA in males and post-menopausal females, it is recommended that they undergo testing for celiac disease and *Helicobacter pylori* along with having bidirectional endoscopy to assess for gastrointestinal blood loss, followed by a trial of iron supplements [2]. If an etiology was not found, performing a video capsule endoscopy is warranted to assess for a gastrointestinal pathology causing bleeding, mainly ileal and jejunal as they are not routinely assessed by bidirectional endoscopy [2].

Common culprits causing gastrointestinal bleeds, which in turn lead to IDA, found during bidirectional endoscopies include but are not limited to peptic ulcer disease, vascular malformations, and malignancy [5]. Primary mucosal melanoma is a rare entity representing only 1.3% of all melanomas, it can develop in any portion of the gastrointestinal tract with varying incidences, prognoses, and treatment options based on anatomical location and stage of malignancy [6,7]. Previous case reports have documented finding mucosal melanomas in unusual locations [3,8].

Diagnostic criteria for primary gastrointestinal melanoma versus a more common metastatic melanoma to the gastrointestinal tract have been debated in order to adequately diagnose the entity. Proposed criteria for the diagnosis of primary mucosal melanoma include the absence of concurrent lesions and no history of prior removal of melanoma or atypical melanocytic lesions from the skin or other organs [8,9].

When assessing a diagnosis of melanoma in an extracutaneous location, an in-depth dermatological and ophthalmological evaluation should be performed to assess for a primary source to rule out metastasis, before labeling the melanoma primary to a non-cutaneous site; in addition, histological findings will support the diagnosis [3,10].

Different treatment modalities have been suggested for mucosal melanomas. Most commonly, local tumor excision when applicable, in addition to chemotherapy and radiotherapy tailored along different histological findings and tumor markers, has been the mainstay of treatment when surgery is not appropriate or anatomically not possible with a reported overall three-year survival rate of 44.0% [6,11,12]. 

## Conclusions

Our case report highlights the importance of a thorough investigation when faced with IDA in males and post-menopausal females, as malignancy remains a primary differential diagnosis. It also emphasizes the American Gastrointestinal Association's recommendation of performing bidirectional endoscopy in these patients, which was essential in diagnosing our patient's rare entity of primary gastric mucosal melanoma. The case also underscores the necessity of utilizing different imaging modalities to stage the disease, including PET scan and video capsule endoscopy, as well as the importance of a complete dermatological and ophthalmological evaluation to assess for a primary source.

Furthermore, our report adds to the literature on primary mucosal melanoma, a rare entity with varying incidences, prognoses, and treatment options based on anatomical location and stage of malignancy. The diagnostic criteria for primary gastrointestinal melanoma versus metastatic melanoma to the gastrointestinal tract have also been discussed, highlighting the importance of appropriate evaluation and histological findings to confirm the diagnosis. Ultimately, our report underscores the importance of a multidisciplinary approach to diagnosing and managing these rare malignancies.
